# Infection with high-risk genotypes of human papillomavirus and cervical cytological findings among kidney transplant recipients in Kenya: a single centre experience

**DOI:** 10.4314/ahs.v22i2.11

**Published:** 2022-06

**Authors:** Millicent S Masinde, Joshua K Kayima, Wanyoike J Gichuhi, Eunice C Cheserem, Orora I Maranga, Samuel K Kabinga

**Affiliations:** 1 Obstetrics and Gynaecology Department, Kenyatta National Hospital, P.O. Box 20723-00202 Nairobi Kenya; 2 East African Kidney Institute, University of Nairobi, P.O. Box 30197 - 00100 Nairobi, Kenya; 3 Obstetrics and Gynaecology Department, University of Nairobi, P.O. Box 30197 - 00100 Nairobi Kenya

**Keywords:** Cervical carcinoma, kidney transplant recipients, high risk Human Papillomavirus

## Abstract

**Background:**

High-risk human papillomavirus (hrHPV) infection is linked with uterine cervix premalignant lesions and invasive carcinoma of the uterine cervix.

**Methods:**

Descriptive cross sectional study carried out among female kidney transplant (KTx) recipients in Kenyatta National Hospital, Nairobi-Kenya. We studied the risk factors for acquisition of hrHPV, examined cervical cytology and assayed for 14 hrHPV DNA using Cervista® HPV HR test and Cervista® MTA (Hologic®) automated platforms.

**Results:**

The 14-hrHPV genotypes assayed were 16, 18, 31, 33, 35, 39, 45, 51, 52, 56, 58, 59, 66, and 68 and the prevalence rate was 31.25 % (10/32). Abnormal cervical cytology was noted in 4/32 (12.5%) and included low-grade squamous intraepithelial lesion (2/32), atypical squamous cells of undetermined significance (1/32) and atypical glandular cells (1/32). The average age was 41.9 years with mean age at first coitus being 20.4 years. Majority of the women 20(62.5%) were married while 8(25%) were single. About 18(56.3%) had only one sexual partner. About 20% of women were nulliparous and 4(12.5%) had a parity of five. Duration since transplantation ranged between 1–21 years.

**Conclusions:**

The burden of hrHPV and abnormal cervical cytology in our study seemed lower than that reported elsewhere and even in general population. This study may form basis for further studies about HPV infections and carcinoma of the uterine cervix among the kidney allograft recipients in our setting.

## Introduction

Human papillomavirus (HPV) is a ubiquitous, non-enveloped DNA virus that can infect squamous epithelia of skin and mucous membranes^1^. In stratified epithelia, HPV infects cells in the basal layer, most likely via epithelial wounding or microfissures^2^. Integration of virus DNA into host chromosomes takes place and virus oncogenes E6 and E7are produced. There is expression of oncoproteins E6 and E7 which interact with various host proteins to aid immune evasion and ultimately persistence of infection in the host^3^. This is associated with neoplastic progression including deregulated expression of virus early genes in basal epithelial cells and genomic instability causing secondary host genomic imbalances^4^. The cervical transformation zone at the squamo-columnar junction may be susceptible to malignancy due to the heightened accessibility of epithelial reserve cells or stem cells in this region 5, 6. This is thought to be important in the carcinogenesis.

Human papillomaviruses have been implicated in virtually all cases of carcinoma of uterine cervix 7. Human papillomaviruses have been categorized as high-risk Human Papillomavirus (hrHPV) and low-risk types based on their oncogenic potential and association with carcinoma of the uterine cervix 8. Several genotypes of hrHPV are known to transform the epithelium and result in cancer^9^. Premalignant lesions precede development of invasive carcinoma of the uterine cervix 10.

Exfoliative cervical cytology has been the conventional approach of screening for carcinoma of uterine cervix. Papanicolaou (Pap) smear has relatively low sensitivity for high-grade cervical dysplastic lesions as well as subjectivity in assignment of smears to the diagnostic categories. These limitations have led to the advent of HPV DNA testing as an adjunct to cytology to improve cervical cancer prevention 11, 12.

Carcinoma of the uterine cervix ranks as the fourth most common female malignancy worldwide. It is a major global health challenge and carries 18 times higher mortality in low- and middle-income countries^13^, ^14^. The highest prevalence of HPV-induced cervical cytological changes occurs early in the third decade, while the invasive carcinoma occurs in the sixth decade 10. Abnormal cervical cytology and hrHPV-related disease in patients with previous organ transplantation has shown lower rates of spontaneous regression 15. Some studies have reported significantly high prevalence rates for both hrHPV infection as well as cervical dysplasia in kidney allograft recipients 16–18. Immunosuppression from medications used in kidney transplantation put the recipients at a higher risk for infection with a broad range of hrHPV genotypes which is plausibly hypothesized to translate into higher prevalence of cytological abnormalities than in the general population 19. The increase in kidney transplantation population should alert nephrologists that cancer is likely to increase over time^20^ especially viral-related malignancies. This demands accurate pre-transplant screening, and post-transplant monitoring of viral infections and tailor-made immunosuppressive protocols.

The role for HPV DNA testing is to improve diagnostic accuracy and avoidance of invasive procedures such as colposcopy in patients with borderline or mildly abnormal cytologic test results [9]. Liquid based cytology along with HPV co-testing can improve sensitivity and specificity in screening for cancer of the uterine cervix. This also allows better compliance, as a negative result of both cytology and HPV would allow patients to get a Pap test every 5 years, thereby increasing screening intervals which is economical in resource-limited settings 21.

In Kenya, kidney transplantation started in 1978. There has not been structured screening for HPV or carcinoma of the uterine cervix for the transplanted population. The current study aimed at documentation of the risk factors, burden of the 14-hrHPV infection and cervical cytomorphology in post-kidney transplant female recipients at Kenyatta National Hospital, Nairobi-Kenya in East Africa. The study was registered with the Kenyatta National Hospital — University of Nairobi Ethics and Research Committee, registration number P192/04/2014.

## Methods

### Study design and setting

This was a cross-sectional descriptive study carried out over three months between August and October 2014. It was undertaken in the Renal Department at the Renal Transplant Clinic which runs once a week and the longest clinic revisit period is three months.

### Study population, inclusion and exclusion criteria

From the clinic records, there were 45 post kidney transplant female patients who attended the clinic between August and October 2014. The study recruited all consenting female kidney transplant (KTx) recipients who attended the clinic, were aged between 18 and 65 years and had been on immunosuppressive medications for at least 6 months. Patients with confirmed carcinoma of the cervix were excluded.

### Study procedures

Information about the study was given to all the female clinic attendees as they waited to be attended in the clinic. Informed consent was obtained from eligible participants and a questionnaire was administered to capture age, marital status, age at first coitus, number of sexual partners, parity, duration since transplantation and immunosuppressive regimes.

### Specimens collection and processing procedures

The Pap smear specimen was collected using the standard procedure by one obstetrician gynaecologist. The first cervical brush was used to collect specimen from endocervix and ectocervix by rotating it three to five times. The brush was then rubbed onto the glass slide and specimen fixed with ethyl alcohol before being air-dried. The second brush was dipped in the ThinPrep® specimen bottle and swirled several times and the bottle was then tightly closed. Wet preparation was used to assay the hrHPV DNA. The air-dried slide was immersed in three stains, each of which stains differently for different cells and different parts of a cell. Haematoxylin and Eosin (H&E) stains nuclei blue, Eosin Azure (EA) stains cytoplasm of immature cells as greenish blue, and that of mature cells as pink. Orang-G stains keratin orange in colour. Pap smear results were reported using the Bethesda 2001 system^22^ by a qualified cytologist. The study cytologist had been trained in reporting Pap smears cytology and had more than 15 years of experience in reporting cervical cytology specimens in teaching and referral hospital laboratories. Every 10th slide was re-examined by another cytologist in the teaching and referral hospital for quality control.

For hrHPV DNA assay, the specimens were transported under the conditions prescribed by the manufacturer to Manchester in the United Kingdom for analyses. Human papillomavirus testing of liquid based cytology (LBC) samples was carried out using Cervista® HPV HR test in conjunction with the Cervista® MTA (Hologic®) according to the manufacturer's instructions. This system provides ultra-pure DNA extraction and HPV testing in one sealed unit. Cervista® uses three oligonucleotide mixtures designed to detect 14 hrHPV types within three familial groups based on phylogenetic similarities: Mix 1 detects types 51, 56, and 66; Mix 2 detects types 18, 39, 45, 59 and 68; Mix 3 detects types 16, 31, 33, 35, 52, and 58. A separate human histone 2 gene probe served as an internal control for cellular DNA content within the LBC sample.

A HPV positive signal was indicated by fluorescent signal above an empirically derived cut-off value. A signal to noise value (sample signal measured against signal from a No Target Control) was generated for each of the three mixes and was referred to as HPV Fold-Over-Zero (FOZ). The HPV FOZ ratio was calculated by dividing the highest FOZ value from any one of the three reaction mixtures by the lowest HPV FOZ value of the three mixtures. If the HPV FOZ ratio was equal to or greater than 1.525, the sample was considered positive for hrHPV. Samples with mixed HPV infections resulted in positive signals of similar intensity in two or three reaction wells. Therefore, if the HPV FOZ ratio was lower than 1.525, but the HPV FOZ values in all three mixes were larger than the second cut-off value at 1.93 (default setting), the sample was considered positive for hrHPV in the Cervista HPV HR test [23]. A positive results meant that at least one of the 14 hrHPV DNA was present in the sample. Results obtained from the analyses were appended to the questionnaires. The data were analysed using Statistical Package for the Social Sciences version 21.0 (SPSS®, Chicago).

## Results

**Figure 1:**
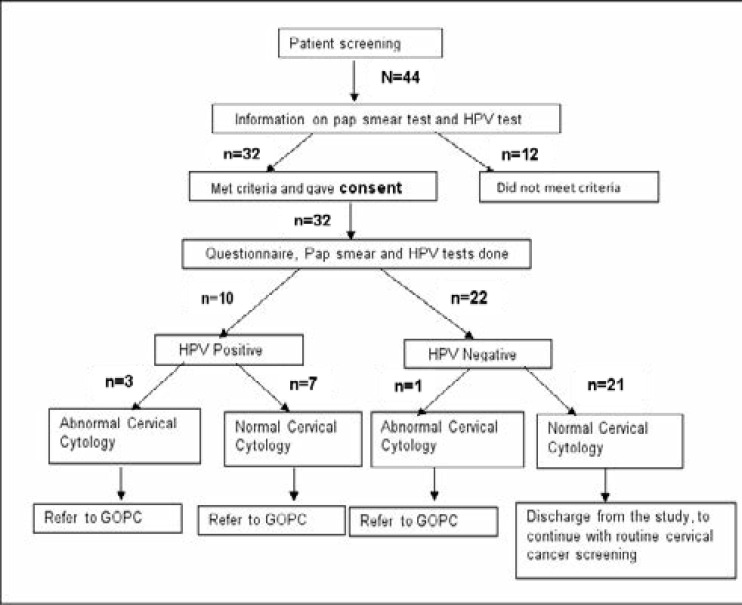
Study participants recruitment flow diagram N: population, n: sample, HPV: Human Papillomavirus, Pap: Papanicolaou, GOPC: Gynaecology Outpatient Clinic

During the period between August and October 2014, 44 of the women who visited the Renal Transplant Clinic for review. Twelve were excluded due to non-eligibility. Thirty two women were enrolled in the study. (Figure 1). Sociodemographic characteristics which included age and marital status were noted. Age at first coitus, number of sexual partners, parity, duration since transplantation and immunosuppressive regimes were analysed. The average age was 41.9±12.9 years with the mean age at first coitus being 20.4±2.7years. Majority of the women 20(62.5%) were married while 8(25%) were single. About 18(56.3%) reported to have had only one sexual partner. One in every 5 women was nulliparous and 4(12.5%) had a parity of five. Duration since transplantation ranged between 1 and 21 years. The commonest triple immunosuppressive regimes were tacrolimus/mycophenolate mofetil/prednisone and cyclosporine/mycophenolate mofetil/prednisone. Azathioprine was used by 3(9.4%) patients in combination with prednisone and either cyclosporine or tacrolimus. About 3 in every 10 women tested had a positive test for at least one of the 14 hrHPV genotype tested (HPV 16, 18, 31, 33, 35, 39, 45, 51, 52, 56, 58, 59, 66, and 68). Four women (12.5%) had abnormal cervical cytology. Only one of the 4 women with abnormal cervical cytology tested negative for hrHPV. (Table 1)

**Table 1:** Sociodemographic and clinical characteristics

Characteristic			p value
Age (year)	Mean±SD	41.9±12.9	**<0.001**† (95 CI, 37.2–46.5)
Age at first coitus (yr)	Mean±SD	20.4±2.7	**<0.001**† (95, CI 19.4–21.4)
Duration since transplantation(yr)Median		5.0	**<0.001**† (95 CI, 3.0–6.8)
Number of sexual partners (number)		% (n)	**<0.001**†(95, CI 1.4–2.0)
	One	56.3 (18)	
	Two	21.9 (7)	
	Three	21.9 (7)	
Parity (number)		% (n)	**<0.001**† (95, CI 1.5–2.7)
	0	21.9 (7)	
	1	18.8 (6)	
	2	25.0 (8)	
	3	12.5 (4)	
	4	9.4 (3)	
	5	12.5 (4)	
Marital Status		% (n)	**<0.001** ‡
	Married	62.5 (20)	
	Single	25.0 (8)	
	Widowed	6.3 (2)	
	Separated	6.3 (2)	
Immunosuppressive regime		% (n)	0.017‡
	CYA/MMF/PRED	37.5 (12)	
	CYA/AZA/PRED	3.1 (1)	
	TAC/MMF/PRED	37.5 (12)	
	TAC/AZA/PRED	6.3 (2)	
	Others	15.6 (5)	
hrHPV		% (n)	<0.052‡
	Positive	31.3 (10)	
	Negative	68.8 (22)	
Pap smear cytology		% (n)	**<0.001** ‡
	Normal	87.5 (28)	
	LSIL	6.3 (2)	
	ASCUS	3.1 (1)	
	AGC	3.1 (1)	

SD: Standard deviation, CYA/MMF/PRED: Cyclosporine/Mycophenolate mofetil/Prednisone, CYA/AZA/PRED: : Cyclosporine/Azathioprine/Prednisone, TAC/MMF/PRED: Tacrolimus/Mycophenolate Mofetil/Prednisone, TAC/AZA/PRED: Tacrolimus/Azathioprine/Prednisone, Pap: Papanicolaou, LSIL: Low-grade Squamous Intraepithelial Lesion, ASCUS: Atypical Squamous Cells of Undetermined Significance, AGC: Atypical Glandular Cells, hrHPV: high-risk Human Papillomavirus

† one-sample T-test

‡ Chi-square test

yr: year

Among the ten women who tested positive for hrHPV, their mean age was 36.1±8.3 years with mean age of first coitus at 21.5±2.6 years. The mean duration since transplantation was 8.2±7.4 years. About 6(60%) were single and 7(70%) had only one sexual partner. Half (5) were nulliparous. The Pap smear cytology showed low-grade squamous intraepithelial lesion (LSIL), atypical squamous cells of undetermined significance (ASCUS) and atypical glandular cells (AGC) in one woman each. (Table 2).

**Table 2:** Characteristics of the ten kidney transplant recipients with positive hrHPV

Characteristic		Results
Age (year)	Mean±SD	36.1±8.3
Age of first coitus (year)	Mean±SD	21.5±2.6
Years since kidney transplantation	Mean±SD	8.2±7.4
Marital status		% (n)
	Married	20 (2)
	Single	60 (6)
	Separated	20 (2)
Number of sexual partners		% (n)
	One	70 (7)
	Two	20 (2)
	Three	10 (1)
Parity		% (n)
	Nulliparous	50 (5)
	1	20 (2)
	2	30 (3)
Pap smear results		% (n)
	Normal	70 (7)
	LSIL	10 (1)
	ASCUS	10 (1)
	AGC	10 (1)

Pap: Papanicolaou, LSIL: Low-grade Squamous Intraepithelial Lesion, ASCUS: Atypical Squamous Cells of Undetermined Significance, AGC: Atypical Glandular Cells, hrHPV: high-risk Human Papilomavirus

## Discussion

Human papillomavirus types have been classified as carcinogenic or probably carcinogenic (group1 or 2A) to humans by the International Agency for Research on Cancer Working Group on the Evaluation of Carcinogenic Risks to Humans 24. Certain HPV are referred to as high-risk types due to their carcinogenic potential to humans 25, 26. The hrHPV positivity rate has been reported to increase as the severity of uterine cervix cytological lesions increase in the general population^1^.

Our study examined the exfoliative cervical cytomorphology and assayed 14 hrHPV genotypes which included 16, 18, 31, 33, 35, 39, 45, 51, 52, 56, 58, 59, 66, and 68. We also explored risk factors for acquisition of HPV and development of carcinoma of the uterine cervix among the female kidney allograft recipients. About a third (31.5%) of our study participants tested positive for at least one of the 14 hrHPV assayed. This compares with the burden of hrHPV reported in North India at 32.5% among KTx recipients^27^.

The risk factors for development of cancer of the cervix are those associated with acquisition of HPV and severe impairment of immune response to HPV infection 28 Solid organ transplantation is a known risk due to long term use of immunosuppressive medications. There have been conflicting reports about the burden of hrHPV infections among kidney transplant recipients with some studies reporting prevalence rates of almost 60% 29, 30 while others have reported rates similar to the general population 31, 32. There have been inconsistent reports on the prevalence rates of infection with hrHPV among kidney allograft recipients. Some studies have documented low and others high prevalence rates 31, 33–35. In kidney allograft recipients, prevalence rates of hrHPV infection from 5% to 53% has been reported^31^, ^32^, ^36^, ^37^. In Columbia, among non-vaccinated women aged 18–25 years, the prevalence of HPV positivity was 60.3%^38^. Among women in general population in Ghana, Krings et al reported prevalence of 32.3% for single hrHPV 39. In coastal town of Kenya, Vuyst et al reported prevalence rate 42.3% of 33 common HPV types among women aged above 15 years 40.

There have been reports of increased rate of cervical cytological abnormalities post kidney transplantation 41. This is thought to be due to prolonged persistence of virus due to impaired clearance by the immune system which is unique to solid transplant recipients due to iatrogenic immunosuppression. Marked improvement in immediate post-transplant care partly due to use of multiple potent immunosuppressive therapies, increase in life expectancy after kidney transplantation theoretically can play a part in acquisition of the viruses and even development of other post-transplant malignancies^31^, ^35^. The commonest triple immunosuppressive regimes were tacrolimus/mycophenolate mofetil/prednisone and cyclosporine/mycophenolate mofetil/prednisone. Azathioprine was also used in combination with prednisone and either cyclosporine or tacrolimus. The duration and drug dose combinations used are reported as important in the development of HPV infection. This was equally observed by Origani et al^36^, Aggarwal et al^27^ and Meuwiss et al^35^, who observed a correlation between the use of immunosuppressive drugs and occurrence of cervical cytological abnormalities.

Abnormal cervical cytology was noted in 4/32 (12.5%) women although one of the women had abnormal cytology but tested negative for any of the 14 hrHPV assayed. The abnormal Pap smear cytology found were low-grade squamous intraepithelial lesion (LSIL), atypical squamous cells of undetermined significance (ASCUS) and atypical glandular cells (AGC) in equal proportions. Global prevalence of HPV infection in women with no cervix abnormalities is 11–12%, with sub-Saharan Africa having a prevalence rate of 24%^42^. In our case, 7/10(70%) of the post KTx recipients tested positive for hrHPV but had normal cervical cytology.

Multiple sexual partners, early sexual debut, non-attendance for screening and under screening are recognized traditional risk factors for acquisition of hrHPV and subsequent development of cancer of the cervix 43, 44. More than 40% of the participants had multiple sexual partners. About 63% of them reported to have had their first coitus at age 14 – 21 years. Majority were married with only a quarter being single. About 6 in every 10 of the kidney transplant recipients reported a parity of 1–3, with more than a fifth being nulliparous. Among the 4 patients who had abnormal cytology, one who had LSIL tested negative for hrHPV.

## Conclusion

The burden of hrHPV and abnormal cervical cytology in our study seemed lower than that reported elsewhere and even in general population. It is plausible to attribute this to lifestyle of patients with end stage kidney disease who undergo transplantation. They are likely to be more health-conscious.

Our study had several limitations. The observational cross-sectional design with no follow up and relatively small sample size. Genetics, viral persistence and environmental factors which can predispose to abnormal cervical cytomorphology other than HPV infections were not studied. This therefore makes it unable to attribute causes and effects to iatrogenic immunosuppression. It is difficulty to generalize its findings to other populations. However we believe these findings can form basis for other studies in this field.
